# Bedaquiline has potential for targeting tuberculosis reservoirs in the central nervous system†

**DOI:** 10.1039/c8ra00984h

**Published:** 2018-03-28

**Authors:** Annapurna Pamreddy, Sooraj Baijnath, Tricia Naicker, Sphamandla Ntshangase, Sipho Mdanda, Hlengekile Lubanyana, Hendrik G. Kruger, Thavendran Govender

**Affiliations:** Catalysis and Peptide Research Unit, School of Health Sciences, University of KwaZulu-Natal Westville Campus Durban 4000 South Africa govenderthav@ukzn.ac.za

## Abstract

Bedaquiline (BDQ) is the first-in-class United States Food and Drug Administration (US FDA) approved anti-tuberculosis (anti-TB) drug, which is a novel diarylquinoline antibiotic that has recently been utilized as an effective adjunct to existing therapies for multidrug-resistant tuberculosis (MDR-TB). BDQ is especially promising due to its novel mechanism of action, activity against drug-sensitive and drug-resistant tuberculosis (TB) in addition to having the potential to shorten treatment duration. Drug delivery to the central nervous system (CNS) is a major concern in TB chemotherapy, especially with the increasing cases of CNS-TB. In this study, we investigated the CNS penetration of BDQ in healthy rodent brain. Male Sprague-Dawley rats (*n* = 27; 100 ± 20 g) received a single 25 mg kg^−1^ b.w dose of BDQ *via* intraperitoneal (i.p.) administration, over a 24 h period. Liquid chromatography-tandem mass spectrometry (LC-MS/MS) was used to determine whole tissue drug concentrations and matrix-assisted laser desorption/ionization mass spectrometry imaging (MALDI MSI) was utilized to evaluate drug distribution in the brain. BDQ reached peak concentrations (*C*_max_) of 134.97 ng mL^−1^ in the brain at a *T*_max_ of 4 h, which is within the range required for therapeutic efficacy. BDQ was widely distributed in the brain, with a particularly high intensity in the corpus callosum and associated subcortical white matter including the striatal, globus pallidus, corticofugal pathways, ventricular system, basal forebrain region and hippocampal regions. Using MALDI MSI, this study demonstrates that due to BDQ's distribution in the brain, it has the potential to target TB reservoirs within this organ.

## Abstract 2

Bedaquiline (BDQ) was administered to healthy Sprague-Dawley rats in order to determine its localisation in the brain using mass spectrometry imaging (MSI). This study shows that BDQ has the potential for targeting TB reservoirs in the CNS.

## Graphical abstract

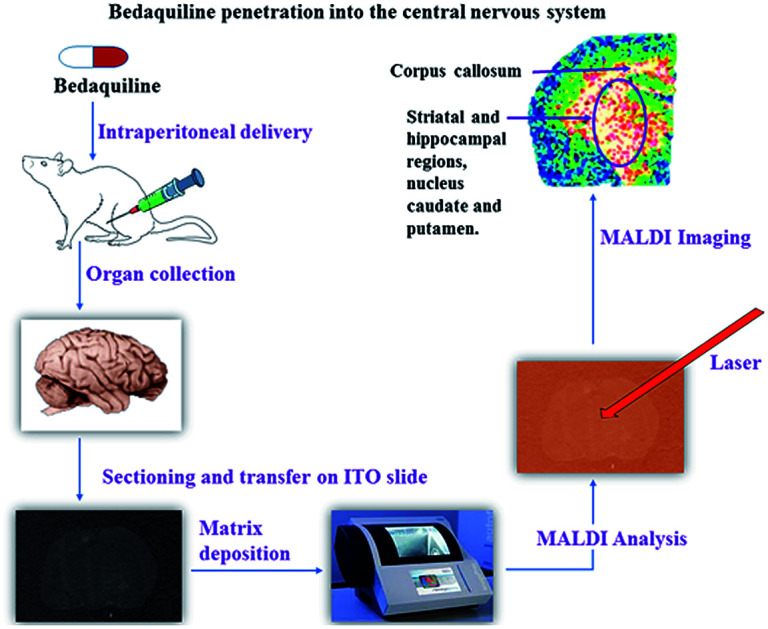

## Introduction

In 2016, the World Health Organization (WHO) estimated 10.4 million tuberculosis (TB) cases and 1.7 million related deaths, among them 1.4 million (10%) were co-infected with HIV.^1^ In the most recent TB drug pipeline, there are several novel agents, many of which are currently undergoing clinical trials to determine their potential against TB.^2–8^ Amongst them, BDQ has proven to be a promising first-in-class US FDA approved anti-TB agent, which belongs to the diarylquinoline class of antibiotics. BDQ is proposed for the treatment of multidrug-resistant tuberculosis (MDR-TB) in combination with other active anti-TB drugs, when an effective therapy regimen could not be established.^9,10^ It exhibits excellent *in vitro* activity against *Mycobacterium tuberculosis* (*M.tb*) isolates that are resistant to the present first- and second-line TB drugs.^11,12^ The unique and potent anti-mycobacterial activity of BDQ involves the inhibition of the proton pump of the mycobacterial ATP synthase.^12–14^ Current regimens for drug-resistant TB are expensive, lethal, require extended periods of usage, and are less effective than treatments that are available for drug-susceptible TB. In 2012, Rouan *et al.* confirmed that BDQ offers great potential in shortening the time of current regimens, as well as being a prospective chemotherapeutic agent for MDR/XDR-TB (extensive drug-resistant tuberculosis), by investigating its pharmacokinetic and pharmacodynamic (PK-PD) properties in a murine model of TB.^15^

The development of extra-pulmonary forms of the disease, more specifically infections of the central nervous system (CNS) is one of the major therapeutic challenges in TB treatment.^16^ The entry of foreign substances into the brain is controlled by the blood brain barrier (BBB), which permits only small amounts of the drug to enter, creating a microenvironment where *M.tb* can replicate under sub-optimal drug levels leading to the development of further resistance.^16^ The central nervous system TB (CNS-TB) infection accounts for the highest mortality when compared to other extra-pulmonary forms of the disease.^17–19^ Reports suggest that the global estimate of EP-TB ranges between 17% to 52% of all TB cases.^20^ Globally, EP-TB represented 15% of the 10.4 million cases reported in 2016.^1^ Intracranial TB manifests itself in many ways, including TB meningitis, intracranial tuberculomas, tubercular encephalitis, and brain abscesses.^21^ Currently, limited data regarding effective treatment regimens of drug-resistant forms of the disease in the CNS are available. Despite evidence-based guidelines for the treatment of intracranial TB, the mortality rate remains significantly high.^17–19^ This is mainly due to inadequate data regarding the penetration and localization of anti-TB drugs in the brain. Recently, our research group studied the localization and bio-distribution of TB drugs (clofazimine, linezolid, rifampicin, pretomanid, TBA-354) in the rodent brain.^2–4,6,7,22^ However, these drugs offer partial protection in the CNS, thus further improvements in TB chemotherapy is required.^3,4^

At present, limited data is available on the localization and distribution of BDQ in the brain.^23^ Therefore, a more detailed investigation into its distribution in this organ is necessary. This is further highlighted by WHO guidelines for the use of BDQ, which encourages further evaluation of its properties.^24^ Thus, the main aim of this study was to establish sensitive methods for the quantitation and localization of this drug in the rat brain by using LC-MS/MS and MALDI MSI, in order to better understand its role and mechanism of action in treatment of CNS-TB.

## Materials and methods

### Materials

BDQ was synthesized at CPRU, UKZN (Durban, South Africa) according to previously published methods^25^ and its characterization was in keeping with that reported in literature.^26^ BDQ D6 (ref. 27) was employed as the internal standard (IS). BDQ D6, ethanol, acetonitrile, methanol, formic acid and DMSO (dimethyl sulfoxide) were of analytical grade and purchased from Sigma Aldrich (Munich, Germany). Ultra-pure water was obtained using a Milli-Q purification system from Millipore Corporation (Bedford, MA, USA). IsoFor (isoflurane) was purchased from Safeline pharmaceuticals (Gauteng, South Africa). Hybrid SPE-Phospholipid cartridges (30 mg, 1.0 mL) were supplied by Supelco-Sigma (St. Louis, MO, USA). 4-Hydroxy-α-cyanocinnamic acid (HCCA) and indium tin oxide (ITO) glass slides required for MALDI MSI were purchased from Bruker Daltonics (Bremen, Germany).

### Methods

#### Animal experiments

All of the animal research protocols used in this study was approved by the University of KwaZulu-Natal Institutional Animal Research Ethics Committee (AREC/078/016PD). Male Sprague-Dawley rats weighing between 100 ± 20 g were obtained from the Biomedical Resource Unit (UKZN) and housed under standard conditions of a 12 h light/dark cycle with *ad libitum* access to standard rat feed and water. Animals received an i.p. injection of freshly prepared BDQ (25 mg kg^−1^ b.w) in 10% DMSO and 90% physiological saline solution. Animals (*n* = 3) were then sacrificed at 0.25, 0.5, 1, 2, 4, 6, 8 min and 24 hours post-dose. Animals were euthanized by Isofor (Gauteng, South Africa) overdose and blood collected *via* cardiac puncture in heparinized tubes. Blood samples were immediately centrifuged at 3500 rpm for 10 min and aliquots of plasma (1.5 mL) were stored at −80 °C prior to analysis. Tissues were surgically removed and gradually frozen in liquid nitrogen vapour before storage at −80 °C until analysis.

#### Sample preparation

Brain tissue samples were suspended in 3 volumes of water and homogenized using a manual tissue homogenizer. The following extraction procedure was used to extract BDQ from both plasma and brain samples. An aliquot of 100 μL of spiked sample was prepared with 250 ng mL^−1^ of IS that was added at each quality control (QC) level followed by brief vortexing. 900 μL of methanol was then added to each preparation to allow for the precipitation of the plasma and/or brain homogenate proteins. The mixture was then vigorously mixed for 30 seconds, followed by centrifugation for 10 min at 10 000 rpm at 4 °C. The supernatant was filtered through a Hybrid-SPE-Phospholipid (30 mg, 1.0 mL) cartridge prior to LC-MS/MS analysis.

#### LC-MS/MS analysis

The quantification of BDQ was performed by using an Agilent Technologies 1100 (Agilent, Germany) series HPLC system with an online degasser, gradient pump and an autosampler coupled to a quadrupole-time-of-flight mass spectrometry (Q-TOF-MS) analyser (Maxis-4G, Bruker, Bremen, Germany) fitted with an electrospray ionization (ESI) ion source. The following conditions were used for chromatographic separation. The mobile phases (MP) were ultrapure water with 0.1% v/v formic acid (MP-A) and 100% acetonitrile (MP-B), respectively. Separation was achieved using a YMC Triart C_18_ column (150 mm × 3.0 mm; particle size 3 μm (YMC Co. Ltd, Japan)) fitted with a compatible C_18_ guard column and was operated at room temperature with a mobile phase flow rate of 0.4 mL min^−1^. The LC gradient was initially increased from 5 to 85% MP-B in 8 min, which was then held for 5 min and returned back to 5% of MP-B in 3 min, with a column re-equilibration time of 3 min.

The MS was operated in positive ion mode using a capillary voltage 6000 V and end plate voltage of 500 V, respectively. The nebulizer pressure was set at 1.5 bar and dry gas temperature was 180 °C at a flow rate of 8 L min^−1^ with a mass range of *m*/*z* 200–1200. The ion charge control was set to 200 000 with a maximum accumulation time of 200 ms. The following multiple reaction monitoring (MRM) settings were used to achieve higher sensitivity and selectivity of the method, the isolation widths for BDQ and the IS were 5.0 and 4.0 and amplifications were 31.0 and 30.0. The quantifier ion optimized for the BDQ and IS (BDQ-D6) were 556.5 → 538.5 *m*/*z* and 562.5 → 544.5 *m*/*z*, respectively. Data analysis 4.0 and Quant analysis (Bruker Daltonics, Germany) software's were used to analyze the data.

#### MSI sample preparation

An optimal cutting temperature (OCT) compound was used to obtain serial tissue sections (10 μm thick) from three different animals mid brain (*n* = 3), each tissue section was thaw mounted onto ITO coated glass slides (Bruker Daltonics, Bremen, Germany), using a cryostat (Leica Microsystems CM1100, Wetzlar, Germany) and stored in a vacuum desiccator prior to analysis. Following overnight desiccation, the slides were scanned using a flatbed scanner (HP LaserJet 3055, China). A 7 mg mL^−1^ of HCCA (Bruker, Bremen, Germany) matrix solution in 50% ACN containing 0.2% TFA was prepared, sonicated for 5 min and then transferred to the ImagePrep (Bruker Daltonics, Bremen, Germany). This is a spraying device that automatically deposits matrix solution onto the tissue in a consistent manner under controlled conditions. The total thickness of matrix layer deposited on all slides determined by the optical sensor set at 1.5 V. An optical sensor set at 1.5 V was used to measure the total thickness of matrix layer deposited on all slides. A pre-optimized ImagePrep method was used for the matrix deposition.^28^

#### MALDI MSI analysis

MALDI MSI analysis was conducted on a Bruker Autoflex III Smartbeam (Bruker Daltonics, Bremen, Germany) system, controlled using FlexControl 3.4 (Bruker Daltonics, Germany) acquisition software. The MS method was optimized by spotting BDQ standard solutions onto control brain tissue sections. Both reflectron and LIFT modes were applied for a mixture of standard peptides and standard concentrations of the drug was spotted with HCCA matrix on a ground steel target in a range of *m*/*z* 200–700 was used to calibrate the instrument in positive ion mode. A LIFT method was optimized using 500 laser shots summed up in a random walk pattern using 10 shots at each raster spot from each position, with a laser frequency of 200 Hz, a mass window range of 7 Da and the digitization rate was 0.5 GS s^−1^. All of the tissue sections were imaged with a spatial resolution of 100 μm. A product ion scan of the parent ion (*m*/*z* 556.5) revealed a clear fragmentation pattern with the transition *m*/*z* 556.5 → 538.5 dominating the spectra with collision energy of 60%. An untreated brain section from a control animal was used to identify background signals that may interfere with the analyte detection and to determine a limit of detection, which was done by spotting various concentrations of BDQ (1, 10, 100, 500 and 1000 ng mL^−1^, respectively). The distribution of BDQ was visualized using a parent ion (*m*/*z* 556.5) in three different animal brain sections, while the fragment ion of *m*/*z* 538.5 was used as a qualifier ion. Acquisition was carried out using FlexControl (version 3.4, build 119) and FlexImaging 4.1 (Bruker Daltonics, Bremen, Germany) for the imaging experiments. All spectra were normalized using the window normalization and the IS method that reduces any potential suppression triggered by the matrix during the acquisition process.

## Results

### Pharmacokinetic analysis

The LC-MS/MS quantification method was optimized to determine BDQ concentrations in both biological matrices (plasma and brain) following i.p. administration to male Sprague-Dawley rats. Currently, there is no data available regarding the quantification of BDQ in rat brain homogenate, therefore it is important to develop and optimize a sensitive and valid LC-MS/MS quantitative method. The limit of detection (LOD) and lower limit of quantification (LLOQ) for BDQ was 5 ng mL^−1^ and 25 ng mL^−1^ in both rat plasma and brain, respectively. The mean recoveries of BDQ was evaluated at three QC levels (LQC, MQC and HQC) (ESI Table 2†) and ranged from 97.8 to 103.2% (RSD < 10%). Intra-day and inter-day accuracy and precision for BDQ was determined at three different QC levels by evaluating six replicates. The precision (%RSD) and accuracy of the analyte in plasma and brain tissue was below 10%, all of which were within the limits set by the EMA (ESI Table 3†).

**Fig. 1:**
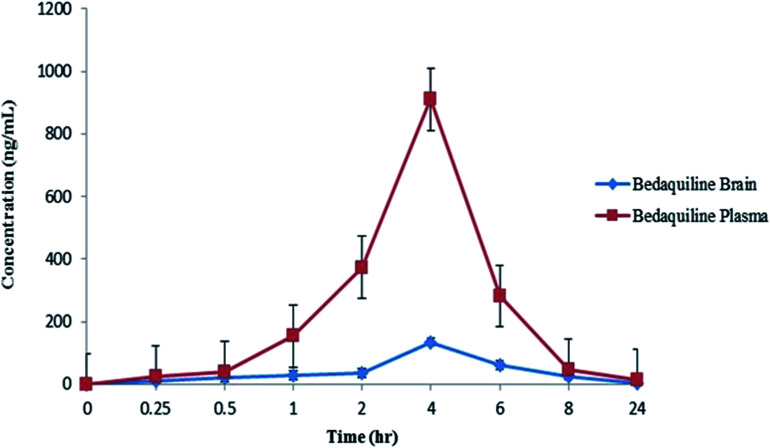
Mean concentration–time profiles of BDQ in plasma and brain tissues following i.p. administration of a single dose of 25 mg kg^−1^ in a rat (*n* = 3). Values are expressed as mean ± SD.

LC-MS/MS quantification has become the gold standard tool for assessing the concentration profiles of a wide range of therapeutics in different biological matrices.^4,6–8,22,28–33^ The optimized method was used to determine tissue pK, following a single dose of BDQ over a 24 h period. Quantitative data (LC-MS/MS) of BDQ in plasma and brain tissues following a single dose (25 mg kg^−1^) of i.p. administration to the rats are shown in Table 1. The plasma concentration of BDQ increased rapidly (Fig. 1), reaching a *C*_max_ of 910.00 ng mL^−1^ at a *T*_max_ of 4 h (Table 1) with a half-life (*T*_1/2_) of 4.99 h and the area under the curve (AUC_0→∞_) was 1034.69 ng h mL^−1^. In Fig. 1, the mean drug concentration *versus* time profiles in plasma and brain homogenates are presented. BDQ reached a *C*_max_ of 134.97 ng mL^−1^ at a *T*_max_ of 4 h in the brain, the area under the curve (AUC_0→∞_) was 362.25 ng h mL^−1^ with *T*_1/2_ of 3.62 h (Table 1) up to 24 h post dose.

**Table 1:** Pharmacokinetic parameters of BDQ in plasma and brain tissues following single dose (25 mg kg^−1^) of i.p. administration in a rat (*n* = 3). Values are expressed as mean ± SD^a^

Parameters	Plasma	Brain
*T*_max_ (h)	4.00	4.00
*C*_max_ (ng mL^−1^)	910.00 (1.40)	134.97 (1.38)
AUC_0→∞_ (ng h mL^−1^)	1034.69 (10.94)	362.25 (6.85)
*T*_1/2_ (h)	4.99	3.62
*K*_e_	0.14	0.19

a Data represents triplicate analysis of both biological matrices, where *n* = 3.

### Mass spectrometry imaging

**Fig. 2:**
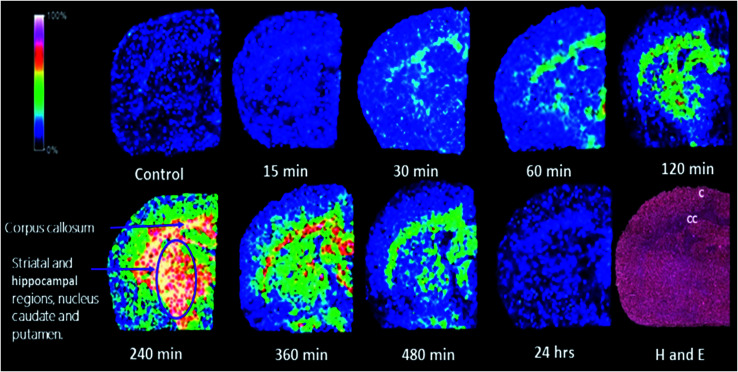
MALDI MSI images of coronal rat brain sections following treatment with BDQ (25 mg kg^−1^ b.w) single dose of i.p. administration, over a 24 h period. MALDI MSI images were acquired using LIFT monitoring *m*/*z* 556.5 ± 0.25%. H and E image is used for histological correlation to the respective brain regions. (C cortex, CC corpus callosum).

The developed MALDI MSI method for BDQ produced reproducible analyses on both ground steel and spotted tissue sections. The LOD was determined by spotting serial dilutions of the drug and defining the lowest concentration with a signal to noise ratio of ≥ 3; the LOD was found to be 1.0 ng mL^−1^. The BDQ parent ion of *m*/*z* 556.5 ± 0.25% was monitored and used to visualize its distribution in the brain. All acquired images were normalized using the window normalization method. In Fig. 2, typical coronal brain tissue sections from the mid-brain of three different animals treated with BDQ analyzed using LIFT mode with a mass filter of 556.5 + 0.25% *m*/*z* are presented with the corresponding hematoxylin and eosin stain (H&E) image for histological correlation (Fig. 2).

## Discussion

In this study, we examined the distribution of BDQ in plasma and brain of healthy rats after an i.p. administration of 25 mg kg^−1^ dose of BDQ, measured from 0.25 to 24 h post-dose.

The LC-MS/MS technique was applied to study the concentration of BDQ in rat plasma and brain tissues. Using this approach, after a 25 mg kg^−1^ single i.p. dose of BDQ, the maximum plasma concentration reached was 910.00 ng mL^−1^ with a *T*_max_ of 4 h, which correlates with previous results that described a *T*_1/2_ of 4 h in the murine models, following a single oral dose of 25–30 mg kg^−1^ of BDQ.^15,34^ The concentration then decreased until no detectable levels of BDQ were evident in plasma after 24 h. It was previously reported^35^ that BDQ has long terminal elimination half-lives (*T*_1/2_ term) in plasma proving its slow release from tissues. The long *T*_1/2_ term of this drug (which accumulates in tissue during multiple dosing in various non-human species) is probably due to a slow release of the compound from peripheral tissues owing to the amphiphilic characteristics (large part of the molecule is lipophilic while the tertiary amine is cationic) of these compounds.^35^ BDQ entered the brain, reaching a *C*_max_ of 134.97 ng mL^−1^ at 4 h of *T*_max_ after i.p. administration. In brain homogenates, the concentration of the drug was detectable up to 8 h and *T*_1/2_ was found to be 3.62 h. All of the concentrations were well within the MIC range of BDQ, which is between 4 to 130 ng mL^−1^ for *M.tb*.^36^

MALDI MSI has been used to effectively determine the accumulation of anti-TB drugs in various tissues.^2–4,6,7,22,37,38^ Studies by Prideaux *et al.* have investigated the penetration of TB drugs into the caseous lesions of the lung.^37,38^ Our group focused on utilizing MALDI MSI to investigate the distribution of anti-TB drugs such as clofazimine, linezolid, rifampicin, pretomanid and TBA-354 in the brain.^2–4,6,7,22^ These studies have shown that different drugs display different distribution patterns. We have also explored the possibility of dissemination of TB bacilli into the brain *via* an aerosol TB infection.^3^ The CFUs of post-treated brain and lung tissues were determined together with drug concentrations found within the serum, lung and brain. Clofazimine displayed a strong bactericidal effect in the lung, whereas linezolid had a bacteriostatic effect.^3^ A recent retrospective study suggested that linezolid may improve outcomes in childhood TBM,^39^ however future controlled studies are needed to validate these claims. Newer anti-TB drugs such as delamanid and bedaquiline may hold promise in this regard, especially in cases of drug resistance.^40^ Currently, no data has been published on pharmacokinetics of delamanid in the brain or CSF, and only one recent case report suggests that BDQ has very poor CSF penetration in an adult diagnosed with TBM.^41^ Therefore it is necessary to investigate the distribution of all potential anti-TB agents to determine the most effective treatment of CNS TB. An FDA advisory^23^ mentioned that with oral administration, BDQ is able to rapidly penetrate tissues due to its cationic amphiphilic drug (CAD) properties.^42,43^ This allows widespread distribution in the adrenal glands, lung, spleen, liver, lymph nodes, thymus and fat, however brain uptake is low,^23^ which lead to the investigation of i.p. administration in this study.

In this study, we successfully used MALDI MSI as a new tool for molecular histology, in order to investigate the localization of BDQ in healthy rat brain sections, following i.p. administration of a 25 mg kg^−1^ b.w dose. We observed the time-dependent distribution of BDQ in the brain, as evidenced by the intense white and pink areas presented on the coloured scale images (Fig. 1). At 0.5 h post dose *via* i.p. administration, the drug was readily detected in the corpus callosum of the brain and suggests that the drug began to permeate the adjacent brain areas, resulting in its widespread distribution at about 1 h post dose. A similar trend of distribution was observed at 2 h post dosage with widespread distribution in the brain and higher intensities in the mesocorticolimbic system, including the cortex, caudate putamen and ventral pallidum regions. At 4 h post dose, the drug diffused into the corpus callosum and associated subcortical white matter, striatal, globus pallidus, corticofugal pathways, ventricular system, basal forebrain region and hippocampal regions which includes the nucleus caudate, putamen and the upper cortex, respectively. The intensity of the drug decreased in the above-mentioned areas and remained low, but persisted in the corpus callosum and striatal-hippocampal region at 6 h post dose. The images depict noticeable elimination of the drug after 8 h, followed by an almost complete decline in drug levels almost to zero at 24 h post dose. There is an excellent correlation exists between the drug concentrations observed between LC-MS/MS quantification data and images acquired using MAlDI MSI technique.

## Conclusions

Our study proved that LC-MS/MS and MALDI MSI can be used as complementary methods for preclinical applications, especially for studies investigating drug distribution in the brain. This is the first study to demonstrate the distribution of a diarylquinoline in healthy rat brain tissues using MALDI MSI. The pharmacokinetics and distribution properties of BDQ reported in this paper prove that the drug has excellent potential in targeting TB reservoirs in the CNS. The use of uninfected rats in this study to determine the concentration and distribution of BDQ in the brain paves the way for future application in an infected animal model.

## Conflicts of interest

The authors report no conflicts of interest. The authors alone are responsible for the content and writing of this article.

## Supplementary Material

RA-008-C8RA00984H-s001
