# Supplementary material for: Bedaquiline has potential for targeting tuberculosis reservoirs in the central nervous system
Source: RSC Adv. 2018 Mar 28;8(22):11902–7. doi: 10.1039/c8ra00984h (PMC9079262; doi:10.1039/c8ra00984h)
Supplement: RA-008-C8RA00984H-s001 [file RA-008-C8RA00984H-s001.pdf]

Electronic Supplementary Material (ESI) for RSC Advances.
This journal is © The Royal Society of Chemistry 2018
Please do not adjust margins

Journal Name

ARTICLE

Supplementary information:

Received 00th January 20xx,
Accepted 00th January 20xx

DOI: 10.1039/x0xx00000x

www.rsc.org/

Bedaquiline has potential for targeting Tuberculosis reservoirs in the central nervous system

Annapurna Pamreddy[a], Sooraj Baijnath[a], Tricia Naicker[a], Sphamandla Ntshangase[a], Sipho Mdanda[a], Hlengekile Lubanyana[a], Hendrik G. Kruger[a], Thavendran Govender[a]*

Method Development

The method development and validation process were done according to the criteria outlined in the EMA guidelines. [1] The following criteria were evaluated; linearity, lower limit of quantitation (LLOQ), limit of detection (LOD), extraction recovery, precision and accuracy.

Stock standards and quality control preparation

Stock solutions (1 mg/mL) of Bedaquiline (BDQ) and IS were prepared in 100% ACN and stored at 4ºC. BDQ working solutions were prepared to a final concentration of 1 µg/mL in ACN:H_2O (50:50). IS working solution was prepared to a final concentration of 1 µg/mL ACN:H_2O (50:50) and stored at 4ºC. Calibration standards and QC samples were prepared using drug free rat plasma or brain samples obtained from BRU, University of KwaZulu-Natal (Durban, South Africa). Calibration standards were prepared in 100 µL plasma or brain homogenate spiked with the working solution containing IS to the final concentration of 100 ng/mL for BDQ. The calibration curve was acquired over the compound concentration range of 10-1250 ng/mL for both brain and plasma, respectively. Similarly, low quality control (LQC), middle quality control (MQC) and high quality control (HQC) levels were prepared in untreated rat plasma or brain homogenate to a final concentration of 30, 300 and 1150 ng/mL for BDQ.

[a.] *1Catalysis and Peptide Research Unit, School of Health Sciences, University of KwaZulu-Natal, Westville Campus, Durban 4000, South Africa.*

*Corresponding author: Professor Thavendran Govender , Catalysis and Peptide Research Unit, School of Health Sciences, E-block, 6th floor, Room E1-06-016, University of KwaZulu Natal, Westville Campus, Durban 4000, South Africa. Offices +27 31 260 1845/1799, Cell +27 73 262 5616,
Email address: govenderthav@ukzn.ac.za.
Electronic Supplementary Information (ESI) available: [details of any supplementary information available should be included here]. See DOI: 10.1039/x0xx00000x

LC-MS sample preparation

An aliquot of 100 µL of plasma or brain homogenates were spiked with BDQ (to a final concentration of 500 ng/mL) and 900 µL of methanol containing IS solution (to a final concentration of 250 ng/mL). Then, the mixture was vortexed for 30 sec, and centrifuged at 10000 rpm for 10 min at 4°C. The plasma/brain supernatant was loaded onto a 30 mg HybridSPE-Phospholipid cartridge (SUPELCO, Pennsylvania, USA) preconditioned with 1 mL of MeOH (100%). Then, the samples were collected into autosampler vials for LC-MS analysis.

Method Validation

Validation of the method was performed according to the European Medicine Agency (EMA) guidelines on bioanalytical method validation [1] using the following parameters: linearity, lower limit of quantification (LLOQ), limit of detection (LOD), precision, accuracy and extraction recovery. The validated method was applied for the LC-MS/MS analysis of BDQ in rodent biological matrices.

Chromatographic conditions

An Agilent technologies 1100 (Agilent, Germany) series HPLC system with gradient pump and an autosampler coupled to a quadrupole-time-of-flight mass spectrometry (Q-TOF-MS) analyser (Maxis-4G, Bruker, Bremen, Germany) fitted with an electrospray ionization (ESI) ion source, was used for chromatographic separation and detection. A YMC column C_{18} Triart (150 x 3.0 mm and 3 µm particle size) (YMC Europe, Gmbh, Germany) was used at room temperature. An LC-MS/MS method was developed for the quantification of BDQ as shown in **Table 1**.

Please do not adjust margins

Please do not adjust margins

Table 1. LC-MS/MS conditions for the quantification of BDQ

**Parameters**	
Mobile phase A	Milli-Q water (0.1% v/v formic acid)
Mobile Phase B	100% ACN
Multistep gradient	Mobile Phase B<br><br>5% (0-1 min), 95% (1-8 min), 95% (8-13 min) 5% (13-16 min)
Flow rate	0.4 mL/min
Injection Volume	5 µl

Results

BDQ raw data information (chiral HPLC chromatogram and ^{1}H NMR spectra) is shown in Figure1 and Figure 2. The developed LC-MS/MS method was optimized for BDQ and BDQ-D_6, the retention time for both BDQ and BDQ- D_6 was 6.0 min (Figure 3A and 3B). The transitions monitored for BDQ was 556.5→538.5 *m/z*. The limit of detection (LOD) for BDQ was 5 ng/mL in both plasma and brain. The lower limit of quantification (LLOQ) of BDQ was 25 ng/mL in both matrices. The extraction recovery of BDQ was accomplished at three QC levels (LQC, MQC and HQC), in five replicates. In plasma and brain tissue, the mean recoveries of the analytes ranged from 97.8 to 103.2% (%RSD values of less than 10%, as required by the EMA) as shown in **table 2**. Intra-day and inter-day accuracy and precision for BDQ was determined by assaying six replicates at three different QC levels. Intra-day and inter-day precision (%RSD) of the analytes in plasma and brain tissue were below 10% **(Table 3)**, all of which were within the limit set by the EMA. The acceptance criterion for accuracy and precision is for the mean concentration to be within 15% RSD of nominal concentration. The validated method was then applied to the LC-MS/MS analysis of the analytes.

Table 2. Mean recoveries of BDQ in different biological matrices (mean within ± 15%)

		**Plasma**		**Brain**	
Substance	Concentration level	Mean Recovery (%)	RSD (%)	Mean Recovery (%)	RSD (%)
BDQ	LQC	99.66	1.32	100.45	1.32
	MQC	99.80	1.55	98.91	2.20
	HQC	98.61	1.05	102.45	2.05

Table 3. Intra- and Inter-day accuracies and precision for BDQ (%RSD with in ± 10%)

		**Plasma**			**Brain**		
Substance		**LQC**	**MQC**	**HQC**	**LQC**	**MQC**	**HQC**
BDQ	Theoretical concentration (ng/mL)	30	300	1150	30	300	1150
	Intra-day mean concentration (ng/mL)	29.26	297.22	1142.38	29.69	296.74	1145.05
	Accuracy (%)	97.54	99.07	99.34	98.99	98.92	99.57
	RSD (%)	0.81	0.93	0.22	1.18	0.84	0.22
	Inter-day mean concentration (ng/mL)	29.19	297.79	1145.09	29.59	297.51	1144.64
	Accuracy (%)	97.29	99.27	99.57	98.63	99.17	99.53
	RSD (%)	0.61	1.15	1.33	1.38	1.31	1.44

RSD= Relative standard deviation

This journal is © The Royal Society of Chemistry 20xx

Please do not adjust margins

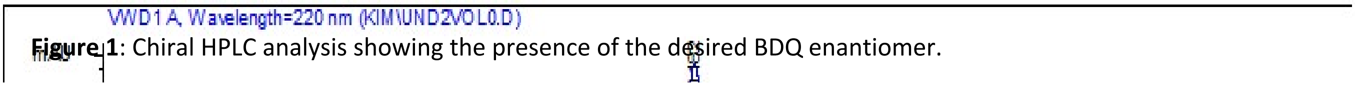

Figure 1: Chiral HPLC analysis showing the presence of the desired BDQ enantiomer.

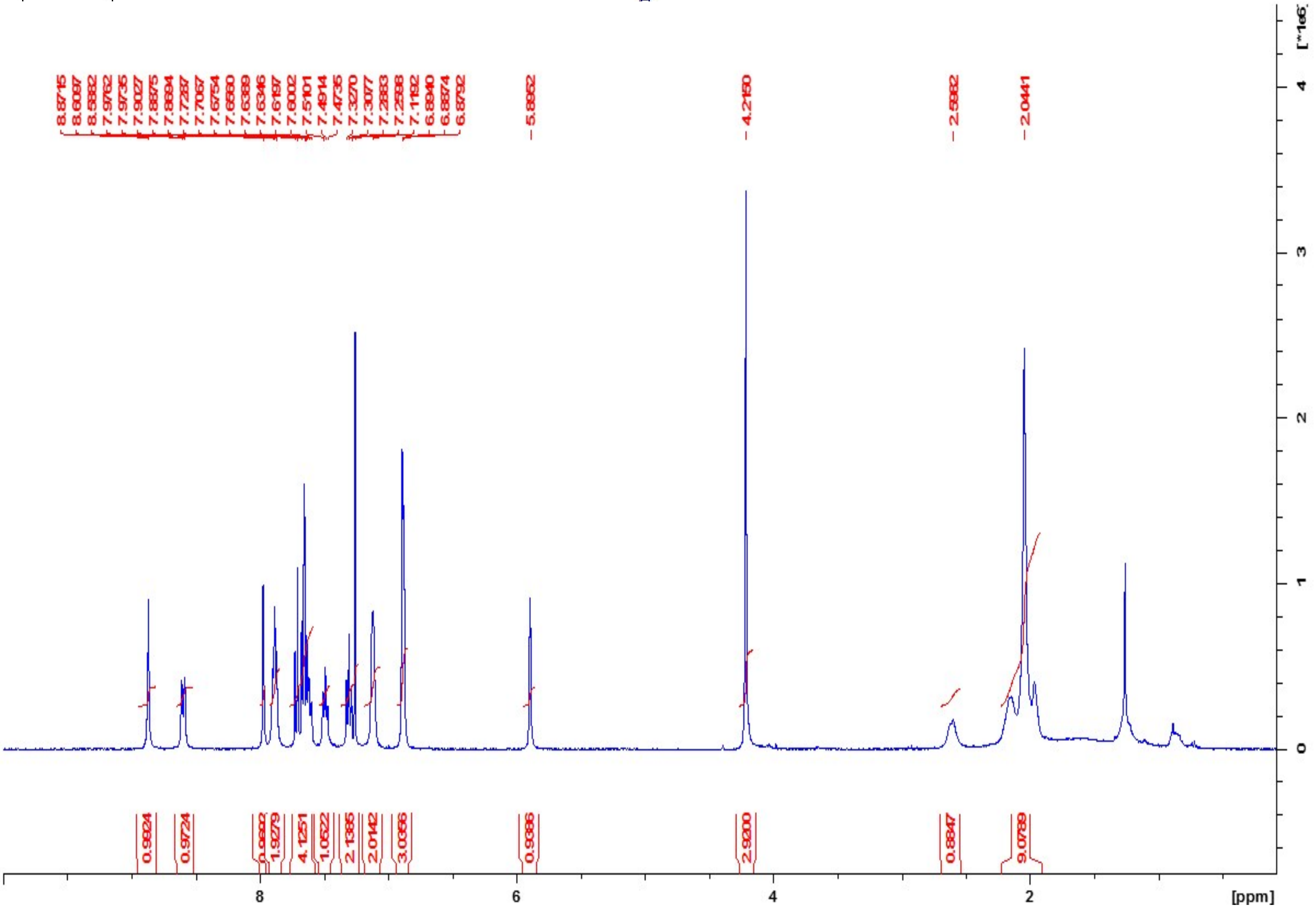

Figure 2: ^{1}H NMR spectra of bedaquiline.

Please do not adjust margins
Please do not adjust margins

Please do not adjust margins

3A

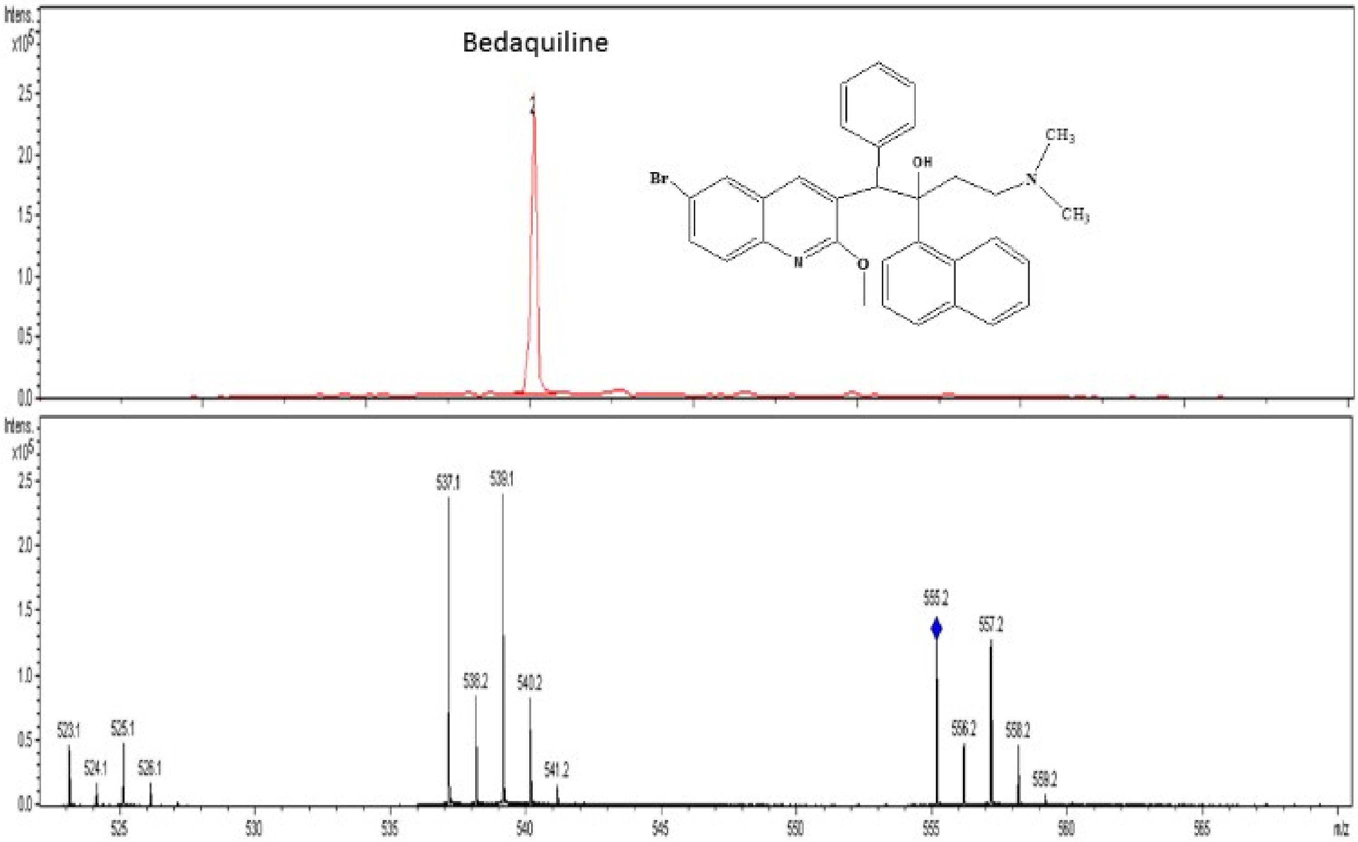

3B

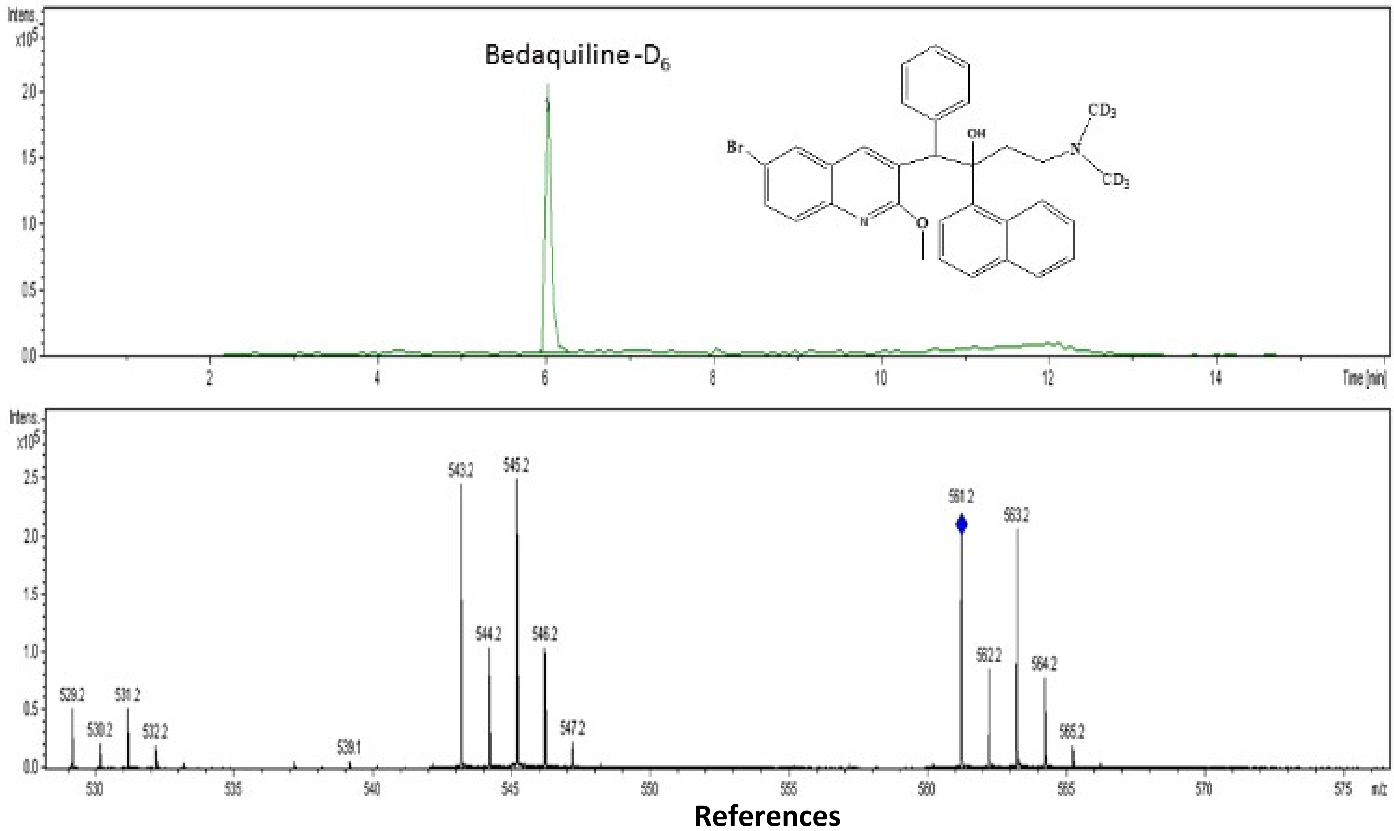

References

 This journal is © The Royal Society of Chemistry 20xx

Please do not adjust margins

Please do not adjust margins

1. European Medicines Agency 2009, C.H.M.P. Committee for Medicinal Products for Human Use. *E. M. A.*, 2009.

This journal is © The Royal Society of Chemistry 20xx

Please do not adjust margins
